# ASO Author Reflections: Double Jeopardy—Risks of Opioid–Benzodiazepine Co-prescription in Older Adults with Gastrointestinal Cancer

**DOI:** 10.1245/s10434-026-19395-6

**Published:** 2026-02-28

**Authors:** Areesh Mevawalla, Timothy M. Pawlik

**Affiliations:** Department of Surgery, The Ohio State University Wexner Medical Center and James Comprehensive Cancer Center, Columbus, OH USA

## Past

Cancer treatment is complex and often involves surgery, systemic therapy, and radiation, each of which can impose substantial physical and psychological burdens.^1^ Patients with gastrointestinal malignancies frequently experience pain, anxiety, insomnia, and other distressing symptoms during treatment, necessitating pharmacologic symptom management.^2^ Opioids and benzodiazepines are commonly prescribed in this setting, sometimes concurrently, particularly among older adults with multiple comorbidities.^3^ Although opioid–benzodiazepine co-prescription has been associated with adverse outcomes in the general population owing to synergistic central nervous system depressant effects, oncology-specific studies have reported mixed evidence regarding whether benzodiazepine co-use confers incremental risk beyond opioid exposure alone.^3,4^ Consequently, the clinical implications of concurrent opioid and benzodiazepine use among older adults with gastrointestinal cancer—especially with respect to prescribing pattern and dose intensity—remain incompletely defined.

## Present

Among 26,896 older adults with gastrointestinal cancer, the median age was 74 years, and 53.1% of patients were female. Overall, 5086 patients (18.9%) were co-prescribed opioids and benzodiazepines during the 12 months following cancer diagnosis. In multivariable time-varying Cox regression models using opioid-only exposure as the reference, opioid–benzodiazepine co-prescription was associated with a higher risk of all-cause hospitalization (hazard ratio [HR] 1.12, 95% confidence interval [CI] 1.03–1.23) and all-cause mortality (HR 1.35, 95% CI 1.06–1.73). When stratified by prescribing pattern, intermittent co-prescription was associated with higher hazards of falls and fractures (HR 1.45, 95% CI 1.08–1.95) and hospitalizations (HR 1.15, 95% CI 1.02–1.30) compared with intermittent opioid-only use, whereas continuous co-prescription (≥ 90 consecutive days) was associated with higher risks of overdose (HR 1.95, 95% CI 1.55–2.45) and mortality (HR 1.35, 95% CI 1.05–1.75) relative to continuous opioid-only exposure. In dose-stratified analyses, the greatest mortality risk was observed among patients receiving high-dose opioid–benzodiazepine co-prescription (≥ 50 morphine milligram equivalents per day).

## Future

Patients with gastrointestinal malignancies often require opioid therapy to manage cancer- and treatment-related pain, particularly during periods of active therapy and functional decline.^5^ In this setting, benzodiazepines may be added to address anxiety, sleep disturbance, or acute symptom exacerbations, especially among older adults with multiple comorbidities.^3^ Adjunctive benzodiazepine use among patients receiving opioid therapy has been associated with incremental risks of hospitalization and mortality.^5^ Recognizing how adjunctive benzodiazepine use interacts with underlying opioid exposure may help clinicians identify patients who warrant closer monitoring or medication review during treatment.^5^ Future studies should further evaluate prescribing patterns and supportive care approaches that balance effective symptom control with the risk of adverse outcomes in older adults with gastrointestinal cancer.
